# Mental health malleability beliefs and help-seeking intentions among Chinese college students: the mediating role of help-seeking self-stigma and the moderating role of depression

**DOI:** 10.3389/fpsyg.2025.1736421

**Published:** 2026-01-12

**Authors:** Wenbo Chen, Junhua Dang

**Affiliations:** 1 Department of Psychology, Beijing Normal University, Beijing, China; 2 Mental Health Education Center, Xihang University, Xi'an, China; 3 School of Humanities and Social Sciences, Xi'an Jiaotong University, Xi'an, China; 4 Department of Surgical Sciences, Uppsala Universitet, Uppsala, Sweden

**Keywords:** beliefs about the malleability of mental health, college students, depression, help-seeking self-stigma, intentions to seek mental health services on campus

## Abstract

**Introduction:**

College years represent a critical period for mental health; however, despite the high prevalence of depression among them, Chinese college students show low utilization of on-campus mental health services. This study examined the associations between beliefs about the malleability of mental health, help-seeking self-stigma, depression level, and intentions to seek mental health services on campus among Chinese college students.

**Method:**

A sample of 2,258 Chinese undergraduates (589 males and 1,669 females, aged between 18 and 25 years old; *M*_age_ = 19.59, *SD* = 1.23) completed questionnaires measuring the core variables, including Implicit Theories of Mental Health Scale, Help-Seeking Self-Stigma Scale, Intentions to Seek Mental Health Services on Campus, and Patient Health Questionnaire. A moderated mediation model was developed to analyze the beliefs about the malleability of mental health, help-seeking self-stigma, depression level, and intentions to seek mental health services on campus.

**Results:**

This study showed that (1) beliefs about the malleability of mental health were positively associated with intentions to seek on-campus services (*b* = 0.44, *p* < 0.001, 95% CI [0.22, 0.66]); (2) help-seeking self-stigma partially mediated this association between beliefs about the malleability of mental health and intentions to seek on-campus services, with an indirect effect value of 0.19, 95% CI [0.15, 0.25] and accounting for 30.51% of the total effect; (3) depression level marginally significantly moderated the first stage of the mediating pathway(interaction term *b* = 0.01, *p* < 0.05, 95% CI [0.0004, 0.02]). Specifically, the negative association between beliefs about the malleability of mental health and self-stigma was weaker among students with higher depression levels.

**Discussion:**

This findings showed that beliefs about the malleability of mental health were positively associated with Chinese college students’ intentions to seek on-campus mental health services by reducing help-seeking self-stigma, and this mediating effect is weakened among students with higher depression levels. These findings provide insights into factors facilitating the utilization of on-campus mental health services and inform targeted interventions to reduce help-seeking self-stigma.

## 1 Introduction

The college years represent a critical developmental period during which students transition from late adolescence to early adulthood, and this stage is characterized by heightened vulnerability to mental health difficulties (Solmi et al., 2022). In China, after enduring the pressures of adolescence and the highly competitive college entrance examination system, many students encounter accumulated psychological distress during their university years (Conteh et al., 2022). Meta-analytic evidence indicates that the prevalence of depressive symptoms among Chinese undergraduates reaches 20.8%, underscoring the substantial mental health challenges faced by this population (Chen et al., 2022). Unaddressed psychological problems can impair academic functioning, disrupt interpersonal relationships, hinder long-term career development, and, in severe cases, contribute to suicidal thoughts or behaviors (Bruffaerts et al., 2018; Buchanan, 2012). Therefore, promoting effective help-seeking among college students is essential not only for alleviating their psychological distress but also for supporting their academic success, socio-emotional adaptation, and overall developmental outcomes.

To improve student well-being, Chinese universities have progressively built campus-based mental health service systems since the mid-1980s (Li and Ma, 2010). In recent years, national policy initiatives have further strengthened these systems. For instance, the *Special Action Plan for Comprehensively Strengthening and Improving Students’ Mental Health Work (2023–2025)*, jointly issued by 17 government departments, mandates that every university establish a mental health counseling center and maintain a counselor–student ratio of no lower than 1:4000 (i.e., at least one full-time counselor for every 4,000 students). These institutional efforts have significantly increased the structural accessibility of mental health services. However, despite the broad availability and cost-free nature of counseling on most campuses, actual utilization remains extremely low. According to the *Report on National Mental Health Development in China (2023–2024)*, only 2.8% of Chinese college students have used on-campus psychological counseling services (Fu, 2025). This striking discrepancy between high mental health need and low help-seeking behavior highlights the importance of understanding why students do not make use of the resources available to them.

Although prior studies have identified many factors associated with students’ hesitation to seek help—such as limited time, inadequate awareness of services, preferences for dealing with problems independently, beliefs that their difficulties are not severe, and concerns about stigma (Lui et al., 2024; Zhao et al., 2025)—these findings do not fully explain the internal cognitive and motivational processes underlying the decision to seek counseling. Help-seeking is not merely a matter of opportunity or awareness; it requires students to evaluate whether their mental health can improve, whether seeking help aligns with their self-perceptions, and whether they have sufficient motivation and psychological resources to act. Thus, understanding students’ willingness to seek mental health services necessitates examining the psychological pathways through which they interpret their difficulties and the potential value of counseling.

Guided by this perspective, the present study focuses on beliefs about the malleability of mental health, which capture students’ belief that mental health problems are changeable and improvable. Such beliefs are foundational in the help-seeking process because they influence whether students perceive counseling as effective and worthwhile (Jin et al., 2023). Prior studies show that individuals who believe their psychological states can change are more motivated to adopt adaptive coping and intervention strategies (Crum et al., 2023). However, even when students believe that change is possible, they may still avoid seeking help if they experience help-seeking self-stigma, defined as internalized negative judgments about receiving psychological support (Vogel et al., 2024). Self-stigma represents an important psychological mechanism through which malleability beliefs may influence help-seeking intentions, as it reflects whether seeking help is viewed as acceptable or threatening to one’s self-image (Mak and Cheung, 2012; Schnyder et al., 2017). Moreover, students’ depressive symptoms may shape the strength of these processes. Depression is known to reduce motivation, weaken self-efficacy, and heighten hopelessness (Acharya et al., 2018), all of which may affect whether malleability beliefs and reduced stigma translate into actual help-seeking intentions. Individuals with higher depression often exhibit help-negation, actively avoiding support even when in need (Chang, 2014; Wilson and Deane, 2010). Thus, depressive symptoms may serve as a meaningful moderator that alters the effectiveness of cognitive and emotional pathways leading to help-seeking.

Based on these considerations, the present study proposes an integrated model in which beliefs about the malleability of mental health predict students’ intentions to seek on-campus counseling services, help-seeking self-stigma serves as a mediator, and depressive symptoms function as a moderator that conditions the strength of these relationships. By capturing the cognitive, evaluative, and motivational stages of the help-seeking decision process, this model aims to clarify why some students are more willing than others to access existing campus mental health services.

Taken together, this study addresses an urgent issue within Chinese higher education: despite substantial institutional investment in mental health service infrastructure, most students in need do not seek professional support. By identifying the internal psychological mechanisms that shape help-seeking willingness, the present research contributes to theoretical models of help-seeking behavior and offers actionable insights for designing interventions that can enhance the accessibility, reach, and effectiveness of university mental health services.

## 2 Literature review

### 2.1 Beliefs about the malleability of mental health and intentions to seek on-campus mental health services

Implicit theories refer to individuals’ fundamental beliefs about whether traits such as their intelligence and personality are malleable, and they can be categorized into two types: entity beliefs (beliefs that traits are fixed) and malleability beliefs (beliefs that traits can be developed; Dweck et al., 1995). Individuals’ adoption of entity or malleability beliefs regarding specific psychological concepts often leads to distinct outcomes. For instance, compared to those who hold entity beliefs of intelligence, students who embrace malleability beliefs of intelligence tend to demonstrate more positive psychological and behavioral outcomes in humans (Dweck and Yeager, 2019). More importantly, malleability beliefs rooted in implicit theories are amenable to intervention. Research has confirmed that interventions targeting malleability beliefs play a facilitative role in fostering positive academic performance and mental health (Crum et al., 2023; Yeager et al., 2022).

People’s mental health is also dynamic and changeable—it may be bad at one stage but good at other times. Therefore, individuals can hold such entity or malleability beliefs of mental health. Jin et al. (2023) extended implicit theories to the domain of mental health, proposing the concepts of implicit theories of mental health. Among these concepts, individuals with entity beliefs of mental health tend to perceive their mental health status as unchangeable, whereas those with beliefs about the malleability of mental health are inclined to take proactive actions to improve their mental health (Jin et al., 2023). Therefore, beliefs about the malleability of mental health are more positive than entity beliefs: they function as protective factors in individuals’ psychological processes and exert a significant impact on their proactive behaviors (Yu et al., 2025). Previous studies have shown that holding beliefs about the malleability of mental health can positively predict proactive coping, cognitive reappraisal strategies, and growth initiative, while negatively predicting negative coping, emotional suppression, and symptoms of anxiety and depression (Jin et al., 2023; Yu et al., 2025). Thus, beliefs about the malleability of mental health may embody a positive orientation of “mental health can be proactively improved,” which can promote individuals to adopt more proactive cognitive and behavioral tendencies to change their mental health trouble as well as a stronger awareness of utilizing mental health resources—such as taking the initiative to seek mental health services on campus.

### 2.2 Mediating role of self-stigma of seeking mental health services

According to the modified labeling theory, self-stigma of help-seeking is one of the most significant barriers that deters people from accessing mental health services (Byrow et al., 2020; Schnyder et al., 2017). Self-stigma of seeking professional psychological help refers to the self-denial and low self-worth experienced by individuals due to their fear of seeking professional psychological help (Vogel et al., 2006). In comparison to public stigma, help-seeking self-stigma exerts a significantly stronger negative predictive effect on an individual’ s help-seeking attitudes and intentions (Yu et al., 2023). Moreover, Self-stigma of help-seeking exhibits greater cultural variability (Chen et al., 2020). Particularly in the context of Chinese culture, seeking help is more likely to be labeled as a sign of “incompetence” or “loss of face” (Mak and Cheung, 2012; Vogel et al., 2024). Consequently, the underlying concern about help-seeking self-stigma among Chinese college students leads them to refrain from accessing campus mental health services when encountering problems.

Notably, individuals with beliefs about the malleability of mental health hold more positive and accurate perceptions of mental health changes (Yu et al., 2025). This cognitive reframing may directly reduce the internalization of self-stigma. Specifically, by decoupling help-seeking from negative self-evaluation, it effectively strengthens college students’ intentions to access campus mental health services. Consequently, Self-stigma of seeking professional psychological help may play a mediating role in the relationship between beliefs about the malleability of mental health and intentions to seek mental health services on campus.

### 2.3 Moderation role of depression

Depression is the most common mental health concern among college students, primarily characterized by persistent sadness, low mood, and diminished interest in activities once found enjoyable (Acharya et al., 2018). Individual depression severity is recognized as a key factor influencing professional help-seeking. For instance, some studies have shown that depression is associated with help-negation, a phenomenon where individuals with mental health needs actively avoid or reject professional support (Chang, 2014; Wilson and Deane, 2010). Specifically, higher levels of depression have been found to negatively predict the likelihood of seeking professional mental health help (Sadek and Awad, 2024; Seidler et al., 2016). Moreover, existing studies further highlight that help-seeking self-stigma serves as a key mediating mechanism underlying this negative association between depression and professional help-seeking (Liang et al., 2022; Özaslan et al., 2024). In other words, greater depression severity may lead individuals to experience higher levels of help-seeking self-stigma, which directly and indirectly reduces intentions to seek help.

Given that beliefs about the malleability of mental health can alleviate help-seeking self-stigma (as noted in previous sections) and promote help-seeking intentions. This path for college students with different severity of depression may be different. That is higher levels of depression among Chinese college students may weaken the predictive effect of beliefs about the malleability of mental health on intentions to seek campus-based mental health services through help-seeking self-stigma. Besides, it remains empirically unclear which stage of this pathway is moderated by depression severity.

## 3 The present study

Building on the theoretical background outlined above, the present study aimed to examine how cognitive beliefs, affective evaluations, and mental health status jointly influence college students’ intentions to seek on-campus mental health services. Prior literature suggests that believing mental health to be malleable may reduce individuals’ internalized stigma toward seeking professional help, thereby increasing their willingness to use available services. At the same time, depression may shape multiple psychological processes involved in help-seeking, including cognitive appraisal, motivational readiness, and self-evaluative judgments. However, existing research does not clearly specify which stage of the help-seeking pathway is most affected by depression.

**Figure 1:**
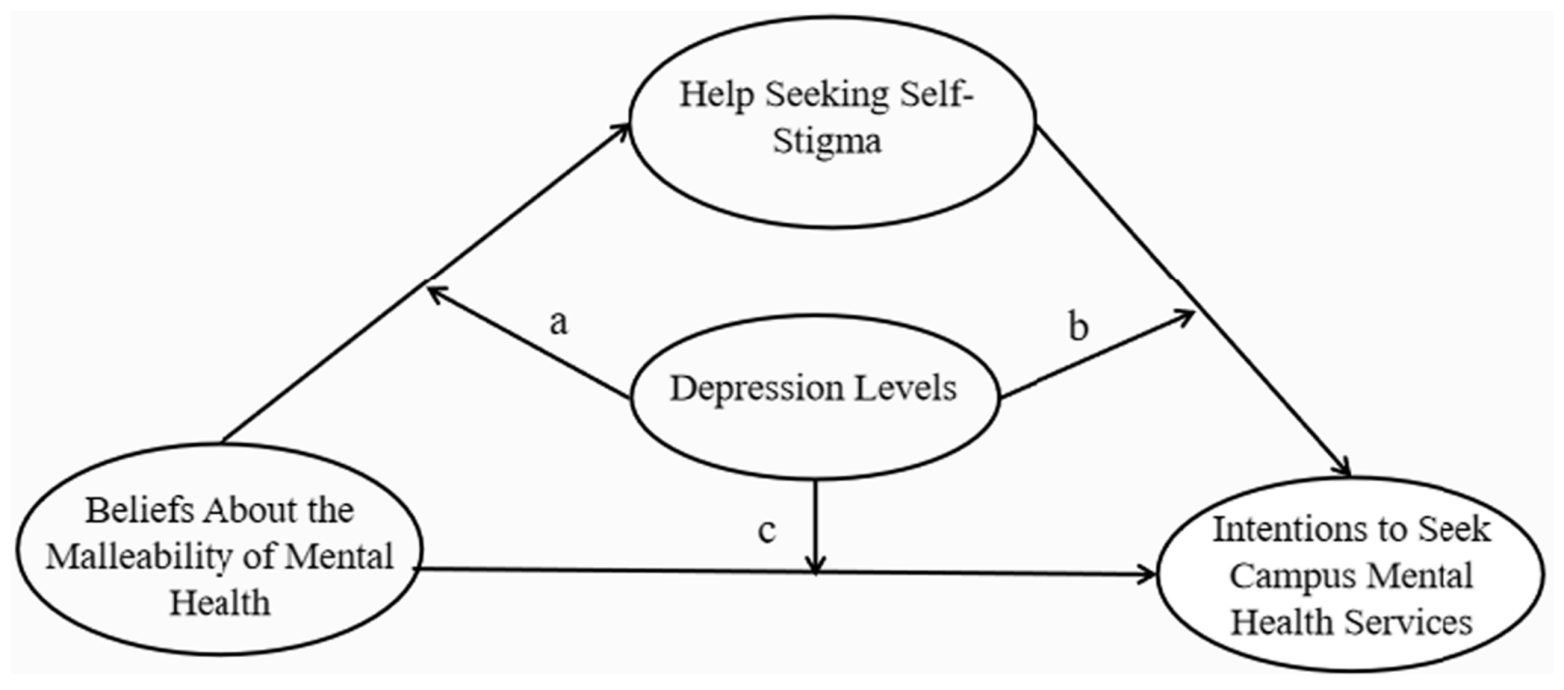
Conceptual framework of the study.

To address this gap, the present study adopted a moderated mediation framework, testing whether the association between beliefs about the malleability of mental health (X) and help-seeking intentions on campus (Y) operates indirectly through help-seeking self-stigma (M), and whether depression (W) moderates any of the underlying structural pathways. Specifically, drawing on theoretical and empirical evidence that depression may influence both cognitive and motivational processes, we examined whether depression moderates (a) the effect of malleability beliefs on help-seeking self-stigma (X → M), (b) the association between self-stigma and help-seeking intention on campus (M → Y), and (c) the direct effect of malleability beliefs on help-seeking intention on campus (X → Y). The hypothesis model were presented in Figure 1. This analytic approach was implemented using PROCESS Model 59 (Hayes, 2018), which allows the moderator to operate on all three paths simultaneously.

Together, this model enables a comprehensive evaluation of the cognitive, affective, and clinical conditions shaping students’ readiness to seek campus-based psychological services. By identifying which components of the help-seeking process are most sensitive to depression severity, the study provides a more refined understanding of how psychological resources and mental health needs interact to influence help-seeking behavior. The findings may inform targeted interventions designed to increase utilization of university mental health services, particularly among students experiencing depressive symptoms.

## 4 Methods

### 4.1 Participants and procedure

This study adopted a cross-sectional design and employed a convenience sampling strategy. College students were recruited from Beijing, Shaanxi, Shandong, Henan, and Guangdong between April and May 2025. Data collection was conducted using Wenjuanxing, a widely used online survey platform in China that is commonly adopted in large-scale psychological and educational research. Questionnaire invitations were randomly distributed to registered student users on the platform.

Prior to participation, all respondents were informed about the purpose of the study, confidentiality protections, and the voluntary nature of participation, and informed consent was obtained electronically. Participants completed the anonymized questionnaire independently in an online setting, without time pressure or researcher supervision. The study procedures complied with the ethical principles outlined in the Declaration of Helsinki for research involving human participants.

Regarding sample representativeness, it should be noted that the use of convenience sampling and an online survey platform may limit the generalizability of the findings. Although Wenjuanxing enables access to a geographically diverse pool of respondents, participants were self-selected and may not fully represent the broader population of Chinese college students. Accordingly, the results should be interpreted with appropriate caution, particularly with respect to population-level prevalence estimates. Nevertheless, this approach is well suited for examining associations among psychological constructs, which was the primary aim of the present study.

With respect to platform reliability, Wenjuanxing provides built-in mechanisms to prevent duplicate submissions (e.g., device and account restrictions) and allows researchers to monitor response patterns and completion time. These features facilitate basic data integrity control and are widely used in peer-reviewed research relying on online survey methodologies in China.

To ensure data quality, standardized data curation and validation procedures were applied prior to analysis. Responses were excluded if they met any of the following criteria: (a) incomplete questionnaire submission; (b) abnormal completion time (less than 60 s or more than 600 s); (c) patterned responding, defined as cyclical repetition across 12 consecutive items; or (d) excessive option repetition, defined as selecting the same response option for eight or more consecutive items and accounting for at least 30% of the total responses. These criteria were applied uniformly across all cases to identify inattentive or careless responding. After data screening, 242 questionnaires were excluded, resulting in a final analytic sample of 2,258 participants, ranged in age from 18 to 25 years (M = 19.59, SD = 1.23). Table 1 presented additional demographic data.

**Table 1:** Demographic characteristics of participants (*N* = 2,258).

Demographic variable	Category	Number (n)	Percentage (%)
Gender	Male	589	26.10
	Female	1,669	73.90
Grade	Freshman	1,322	59.03
	Sophomore	540	23.91
	Junior	305	13.51
	Senior	80	3.55
Registration	Urban	1,538	68.00
	Rural	720	32.00

### 4.2 Measures

#### 4.2.1 Beliefs about the malleability of mental health

The Implicit Theories of Mental Health Scale (ITMH), developed by Jin et al. (2023) with good reliability and validity, was adopted to assess college students’ levels of beliefs about the malleability of mental health. The scale consists of six items, scored on a 6-point Likert scale (1 = strongly disagree, 6 = strongly agree). After reverse-scoring items 1, 2, and 3, the average score of all items was calculated. A higher average score indicates a stronger level of beliefs about the malleability of mental health. The Cronbach’s *α* coefficient of this scale in the current study was 0.77.

#### 4.2.2 Self-stigma of seeking help

The Self-Stigma of Seeking Help Scale (SSHS) was used to measure college students’ self-stigma toward seeking mental health services. Developed by Vogel et al. (2006), this scale has a Chinese revised version adapted by Hao and Liang (2011). The Chinese version self-stigma of seeking mental health services scale includes five items, scored on a 5-point Likert scale (1 = strongly disagree, 5 = strongly agree). A higher score indicates a higher level of self-stigma of seeking mental health services. The Cronbach’s α coefficient of this scale in the current study was 0.92.

#### 4.2.3 Seeking mental health services on campus

A single item of seeking mental health services, adapted from the National Comorbidity Survey (NCS; Kessler, 1994; Murphy et al., 2024), was used to assess college students’ intentions to seek mental health services on campus (ISMHSC): “If you encounter psychological distress that you cannot solve on your own, to what extent are you willing to seek professional help from psychologists or psychiatrists on campus?” The item was scored on a 10-point scale (0 = not willing at all, 10 = very willing), with a higher score indicating a stronger intention to seek mental health services on campus. Because this variable was measured using a single item, internal consistency indices such as Cronbach’s alpha are not applicable. Consistent with previous research in the Chinese context, single-item assessments of help-seeking intention have been widely adopted (e.g., Liu et al., 2021). In the present study, the item was slightly adapted to better reflect the on-campus counseling environment, and the adaptation process has been clarified in the manuscript.

#### 4.2.4 Depression level

The 9-item Patient Health Questionnaire (PHQ-9), originally developed by Kroenke et al. (2001), was used to assess depression levels among college students. The Chinese version validated by Wang et al. (2014) was adopted in this study, as it has demonstrated strong reliability and validity in Chinese populations. Participants responded on a 4-point Likert scale (0 = not at all, 3 = nearly every day), yielding total scores ranging from 0 to 27, with higher scores indicating more severe depressive symptoms. In the present sample, the PHQ-9 showed good internal consistency, with a Cronbach’s *α* coefficient of 0.86.

### 4.3 Data analysis

SPSS 25.0 and the PROCESS macro 3.5 were used for data analysis. The analyses proceeded in three steps: (1) conducting Harman’s single-factor test to examine common method bias; (2) performing descriptive statistics and Pearson correlation analyses among all study variables; and (3) testing the proposed moderated mediation model using PROCESS Model 7, in which depression moderates the first-stage path from beliefs about the malleability of mental health (X) to help-seeking self-stigma (M). Age, gender, grade, and household registration were included as covariates. All continuous predictors were mean-centered before analysis. The bootstrap method with 5,000 resamples was applied to estimate the 95% confidence intervals (CI) for indirect and conditional effects, and effects were considered statistically significant if the CI did not include 0.

## 5 Results

### 5.1 Descriptive analysis

Harman’s single-factor test was used to conduct an unrotated exploratory factor analysis on all scale items. Exploratory factor analysis with unrotated extraction revealed four factors with eigenvalues greater than 1, and the first factor explained 32.94% of the total variance (below the 40% threshold). These results confirm the absence of severe common method bias in the current study. Descriptive statistics (including Means and Standard Deviations) and Pearson correlation analyses were conducted for the core variables, beliefs about the malleability of mental health, self-stigma of seeking professional help, depression level, and intentions to seek mental health services on campus—as well as the demographic variables, age, gender, registration type and grade. As presented in Table 2, beliefs about the malleability of mental health were significantly negatively correlated with self-stigma of seeking professional help (*r* = −0.36) and depression level (*r* = −0.36), while significantly positively correlated with intentions to seek mental health services on campus (*r* = 0.27); Self-stigma of seeking professional help was significantly positively correlated with depression level (*r* = 0.48) and significantly negatively correlated with intentions to seek mental health services on campus (*r* = −0.33); Depression level was significantly negatively correlated with intentions to seek mental health services on campus (*r* = −0.25). Among the demographic variables, several significant correlations with core variables were observed. Age was significantly positively correlated with beliefs about the malleability of mental health (*r* = 0.07) and intentions to seek mental health services on campus (*r* = 0.05), while significantly negatively correlated with self-stigma (*r* = −0.07). Gender was significantly negatively correlated with self-stigma of seeking professional help (*r* = −0.06) and depression (*r* = −0.05), while significantly positively correlated with intentions to seek mental health services on campus (*r* = 0.10). Grade was significantly positively correlated with beliefs about the malleability of mental health (*r* = 0.04) and intentions to seek mental health services on campus (*r* = 0.06), while significantly negatively correlated with self-stigma of seeking professional help (*r* = −0.06).

**Table 2:** Means, standard deviations, and correlations of study measures.

Variables	1	2	3	4	5	6	7	8
1. ITMH	1							
2. SSHS	−0.36**	1						
3. ISMHSC	0.27**	−0.33**	1					
4. PHQ-9	−0.36**	0.48**	−0.25**	1				
5. Age	0.07**	−0.07**	0.05*	−0.03	1			
6. Gender	0.03	−0.06**	0.10**	−0.05*	−0.08**	1		
7. Registration	0.04	−0.02	−0.01	0.003	0.04*	−0.002	1	
8. Grade	0.04*	−0.06**	0.06**	−0.02	0.70**	−0.002	−0.004	1
*M*	4.35	2.15	6.56	6.60	19.59			
*SD*	0.76	0.77	2.11	4.02	1.23			

*p < 0.05, **p < 0.01.

### 5.2 Moderated mediation effect

To test the proposed hypotheses, Model 59 of the PROCESS macro 3.5 was employed, with age, gender, registration type, and grade as covariates. A bootstrap resampling procedure with 5,000 samples was used to assess the significance of indirect effects and 95% bias-corrected confidence intervals (CIs) were reported.

**Figure 2:**
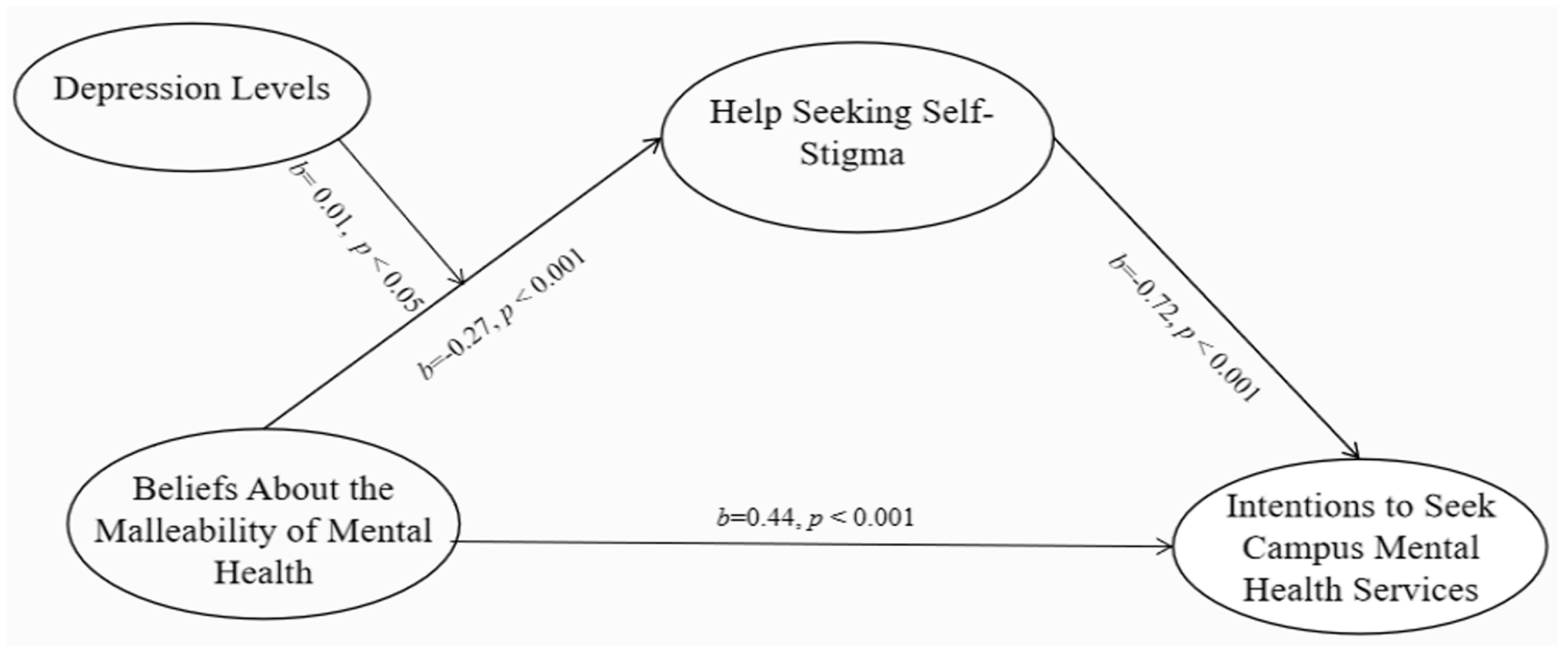
Moderated mediation model of beliefs about the malleability of mental health, help seeking self-stigma, depression, and intentions to seek campus mental health services.

As shown in Table 3 and Figure 2, beliefs about the malleability of mental health negatively predicted self-stigma of help-seeking (*b* = −0.27, se = 0.03, *p* < 0.001); Self-stigma of seeking professional help negatively predicted the intentions to seek campus mental health services (*b* = −0.72, se = 0.11, *p* < 0.001). Meanwhile, the direct effect of beliefs about the malleability of mental health on intentions to seek mental health services on campus was also significant (*b* = 0.44, se = 0.11, *p* < 0.001). These results demonstrate that self-stigma of help-seeking partially mediates the relationship between beliefs about the malleability of mental health and Chinese college students’ intentions to seek campus-based mental health services.

**Table 3:** Regression coefficients testing the moderated mediation.

Predictive variables	SSHS				ISMHSC			
	*b*	se	*t*	*p*	*b*	se	*t*	*p*
SSHS					−0.72	0.11	−6.59	0.000
ITMH	−0.27	0.03	−7.99	0.000	0.44	0.11	3.89	0.000
PHQ	0.04	0.02	2.89	0.004	−0.07	0.07	−0.93	0.353
Age	−0.02	0.02	−1.27	0.205	−0.03	0.05	−0.70	0.482
Gender	−0.06	0.03	−1.98	0.048	0.37	0.10	3.77	0.000
Registration	−0.01	0.03	−0.49	0.622	−0.11	0.09	−1.14	0.253
Grade	−0.02	0.02	−0.76	0.448	0.17	0.07	2.39	0.017
ITMH*PHQ	0.01	0.00	2.06	0.039	0.00	0.01	0.06	0.952
SSHS*PHQ					0.01	0.01	0.71	0.477

**Figure 3:**
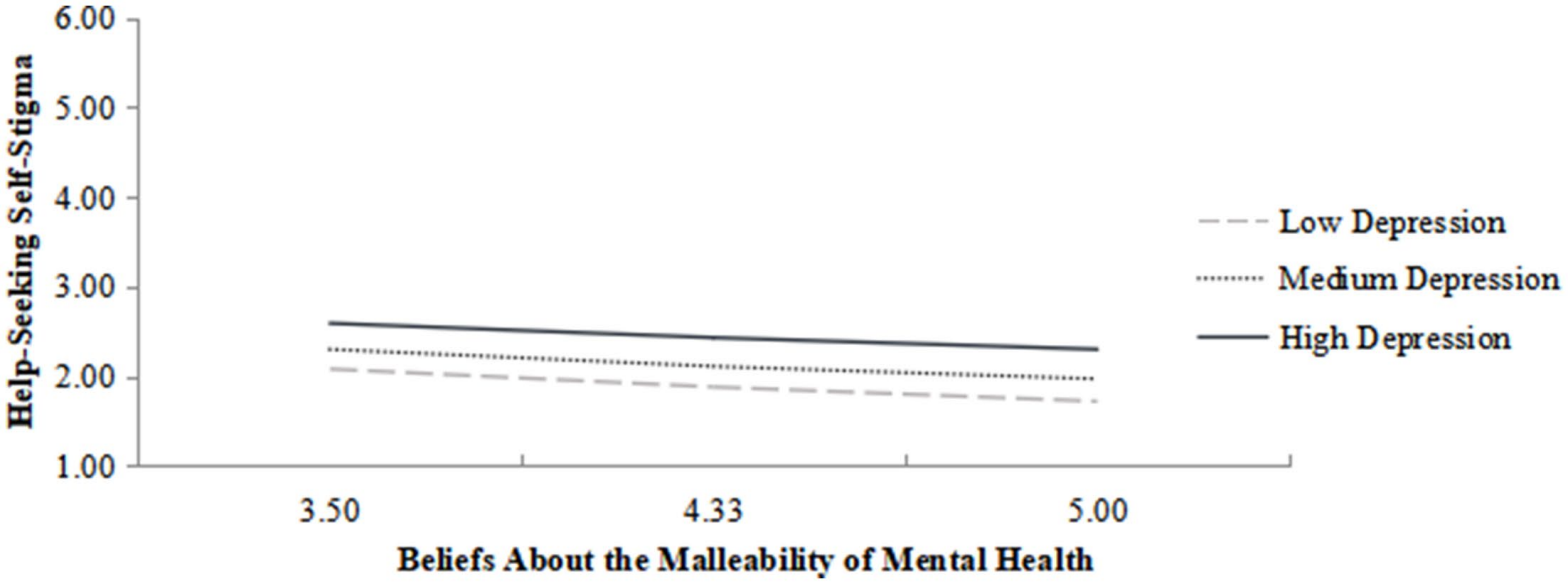
Simple slope analysis of the moderating effect of depression.

The regression model predicting self-stigma of help-seeking showed that the interaction term between beliefs about the malleability of mental health and depression level was statistically significant (*b* = 0.01, se = 0.004, *p = 0*.039). This indicated that depression level significantly moderated the relationship between beliefs about the malleability of mental health and self-stigma of help-seeking (path a). Simple slope analysis was further conducted at three levels of depression (16th percentile: 3.00, 50th percentile: 6.00, 84th percentile: 10.00). As shown in Figure 3, at low depression (3.00), beliefs about the malleability of mental health negatively predicted self-stigma (*b* = −0.24, 95% CI [−0.29, −0.20]). At moderate depression (6.00), the negative predictive effect weakened (*b* = −0.22, 95% CI [−0.26, −0.18]). At high depression (10.00), the negative predictive effect was the weakest (*b* = −0.19, 95% CI [−0.23, −0.15]). Bootstrap analysis confirmed that the indirect effect of beliefs about the malleability of mental health on intentions to seek campus mental health services via self-stigma was significant at all three levels of depression (95% CI excluded 0), and the effect size gradually decreased with increasing depression: At low depression (3.00), indirect effect = 0.17, BootSE = 0.03, 95% CI [0.12, 0.23]; At moderate depression (6.00), indirect effect = 0.14, BootSE = 0.02, 95% CI [0.11, 0.20]; At high depression (10.00), indirect effect = 0.12, BootSE = 0.03, 95% CI [0.07, 0.18].

The regression model predicting help-seeking intentions showed that the interaction term between self-stigma of help-seeking and depression level was not significant (*b* = 0.01, se = 0.01, *p* = 0.477). This suggested that depression level did not moderate the relationship between self-stigma and intentions to seek campus mental health services (path b). Additionally, the interaction between beliefs about the malleability of mental health and depression level on intentions to seek campus mental health services was also not significant (*b* = 0.001, se = 0.01, *p* = 0.950), indicating no moderation of the direct path (path c). Taken together, these results demonstrate that depression level moderates the mediating role of self-stigma of willingness to seek campus mental health services by regulating the first stage of the mediation process. Specifically, the mediating effect is more pronounced among college students with lower depression levels.

## 6 Discussion

Reducing help-seeking self-stigma and promoting the proactive utilization of mental health services are critical pathways toward realizing the the theme of World Mental Health Day of the World Health Organization in 2025 in China for “Everyone Has Access to Mental Health Services”, which has been strongly advocated by the National Health Commission of the People’s Republic of China (NHC). Yet this vision remains far from being achieved among Chinese college students, who exhibit high levels of mental health distress but low rates of professional help-seeking. To address this need–utilization gap, the present study adopted an implicit theory framework to examine how beliefs about the malleability of mental health shape students’ intentions to seek on-campus psychological services. Specifically, we investigated whether help-seeking self-stigma serves as a mediating mechanism, and whether depression severity conditions the strength of these pathways.

### 6.1 Effects of malleability beliefs on help-seeking intentions

Consistent with our hypothesis, students who held stronger beliefs about the malleability of mental health demonstrated greater willingness to seek on-campus mental health services. This finding suggests that perceiving mental health as changeable—and thus improvable—may encourage individuals to adopt more proactive approaches to maintaining psychological well-being (Jin et al., 2023). Within modern university settings where mental health service infrastructure has rapidly expanded, endorsing malleability beliefs may represent a key cognitive resource that increases students’ readiness to seek professional assistance when needed.

Beyond the implicit theory perspective, these results can also be interpreted through Granular Interaction Thinking Theory (GITT). According to GITT, cognitive “information granules” interact with one another to form higher-order value systems (Vuong and Nguyen, 2024). Malleability beliefs may serve as positive granules that interact with existing self-beliefs and sociocultural norms, reinforcing a value system that views help-seeking as adaptive rather than shameful. Similarly, from the Mindsponge Framework (Vuong, 2023)—which emphasizes the subjective cost–benefit filtering of new information—students may selectively absorb malleability beliefs into their core mindset when these beliefs are perceived as beneficial. Once internalized, these beliefs reduce perceived barriers and encourage proactive help-seeking. Integrating these theoretical lenses enriches our understanding of how cognitive beliefs translate into behavioral intentions.

### 6.2 Mediating role of help-seeking self-stigma

This study further found that help-seeking self-stigma significantly mediated the relationship between malleability beliefs and help-seeking intentions. Students with stronger malleability beliefs reported lower levels of self-stigma, and lower self-stigma, in turn, predicted stronger intentions to seek professional help—consistent with extensive research in Western and Eastern contexts (Chen et al., 2014; Clement et al., 2015; Zhao et al., 2025).

From an information-processing view, GITT proposes that cognitive granules dynamically interact; malleability beliefs may weaken stigmatizing cognitive granules (e.g., “seeking help means weakness”), resulting in a more adaptive configuration of values. Likewise, the Mindsponge Framework suggests that when individuals perceive the benefits of help-seeking as outweighing its social or psychological costs, they are more likely to reject stigmatizing beliefs and absorb more facilitative ones. These theories jointly illuminate why malleability beliefs reduce self-stigma and enhance help-seeking intentions, especially in China’s collectivist, face-oriented cultural environment.

### 6.3 Moderating role of depression severity

The moderating effect of depression provides additional insight into the boundary conditions of the malleability belief–stigma–intention pathway. Results showed that depression severity moderated the first stage of the mediating process—i.e., the link between malleability beliefs and help-seeking self-stigma. Among students with higher levels of depression, malleability beliefs were less effective in reducing self-stigma. This aligns with the help-negation literature (Wilson and Deane, 2010), which suggests that depressed individuals may display reduced motivation, lower problem-solving efficacy, and heightened negative self-evaluation—factors that can amplify self-stigmatizing beliefs such as “seeking help is useless” or “seeking help signifies incompetence,” which are salient in Chinese contexts (Maeshima and Parent, 2022; Vogel et al., 2024).

Drawing again on the Mindsponge Framework, depression may distort the subjective filtering of help-related information, increasing the perceived cost of help-seeking and preventing positive beliefs from being integrated into one’s cognitive system. From the perspective of GITT, depressive symptoms may disrupt interactions among cognitive granules, weakening the transformation of malleability beliefs into reduced stigma. Therefore, for students with higher depression levels, the indirect effect of malleability beliefs on help-seeking intentions—via self-stigma—is attenuated. Interestingly, depression did not moderate the M → Y path or the direct X → Y path, suggesting that depression exerts its influence primarily at the cognitive-evaluative (rather than motivational-expressive) stage of the process.

### 6.4 Theoretical, practical, and policy implications

The findings of this study offer a set of integrated implications at the theoretical, practical, and policy levels. Theoretically, the results advance existing scholarship in several meaningful ways. First, by identifying Beliefs About the Malleability of Mental Health as a significant and modifiable predictor of help-seeking intentions, this study extends the implicit theory literature into the domain of mental health service utilization. Previous research on college students in China has predominantly emphasized hindering factors, such as stigma, lack of awareness, and preference for self-reliance; comparatively fewer studies have examined facilitative cognitive resources that motivate help-seeking (Gulliver et al., 2010; Shi et al., 2020). Our findings fill this gap by demonstrating that malleability beliefs—an underexplored cognitive factor—play a central role in shaping proactive help-seeking.

Second, this study provides further support for the modified labeling theory (Schnyder et al., 2017) by confirming that help-seeking self-stigma mediates the relationship between cognitive beliefs and help-seeking intentions. This mediated process is especially meaningful within China’s collectivist and face-oriented cultural environment, where stigma carries substantial social and personal significance (Mak and Cheung, 2012). The results indicate that reducing self-stigma is essential for translating adaptive cognitive beliefs into constructive behavioral intentions.

Third, the moderating role of depression contributes to a more nuanced understanding of boundary conditions within the help-seeking process. The finding that depression attenuates the effect of malleability beliefs on self-stigma suggests that cognitive interventions may be less effective for individuals experiencing higher depressive symptoms. This insight enriches existing cognitive–behavioral models of help-seeking by highlighting differential susceptibility to belief-based interventions depending on clinical symptom levels.

Beyond the primary theoretical frameworks of stigma and implicit beliefs, our findings also resonate with GITT and the Mindsponge Framework, as well as the recently proposed informational entropy–based value formation theory (Vuong et al., 2025). Together, these perspectives emphasize that the mind operates as a dynamic cognitive system in which informational units interact, are selectively filtered, and are assigned value. Within this system, malleability beliefs may function as positively weighted informational granules that facilitate adaptive interpretations of mental health, thereby lowering the negative valuation embedded in self-stigma. Conversely, depression can increase informational entropy and disrupt efficient value formation, leading to a stronger weighting of negative self-relevant information and a reduced capacity to integrate positive beliefs into one’s cognitive system. Integrating these perspectives provides a deeper explanation for why malleability beliefs reduce stigma, why self-stigma reflects a negative valuation of the self, and why depression weakens these adaptive pathways.

Practically, the results offer multiple avenues for improving campus mental health services. Strengthening malleability beliefs through targeted interventions (e.g., growth mindset workshops) could help students develop a more adaptive understanding of mental health and enhance their readiness to seek help. At the same time, stigma reduction efforts—such as anti-stigma campaigns, peer education, and personal narrative sharing—could address emotional and social barriers to help-seeking. Importantly, intervention strategies may need to be stratified by depression severity: students with mild to moderate depressive symptoms may benefit from belief-based interventions combined with stigma reduction, whereas those with more severe depression may require intensive, individualized counseling to restore motivation and perceived efficacy before cognitive interventions become effective.

From a policy perspective, universities and educational authorities may consider embedding malleability belief education into mental health literacy curricula (Yang et al., 2024; Lien et al., 2024), especially during freshman orientation. Policy makers may also prioritize investments in stigma-reduction initiatives and expand counselor availability to ensure adequate service capacity for students with high clinical needs. Such multilevel policies could help narrow the persistent “high need, low utilization” gap (Fu, 2025) in Chinese college settings.

### 6.5 Limitations and future directions

Despite its contributions, this study is subject to several limitations that point to important directions for future research. First, the cross-sectional design limits the ability to make causal inferences about the relationships among malleability beliefs, self-stigma, depression, and help-seeking intentions. Although the proposed moderated mediation model is theoretically grounded, longitudinal or experimental studies are needed to more definitively assess temporal ordering and causal dynamics. Future research may adopt longitudinal tracking of belief changes or implement malleability-focused interventions to test causal effects on stigma and help-seeking behavior (Song et al., 2024).

Second, while depression was measured using the validated Chinese version of the PHQ-9, this instrument is a screening tool and does not provide clinical diagnostic information. Future studies may incorporate structured clinical interviews or clinician-rated assessments to differentiate between subclinical distress and clinically significant depressive disorders. Doing so may reveal whether the moderated mediation model functions similarly across clinical and non-clinical subgroups.

Third, this study examined help-seeking intentions rather than actual help-seeking behaviors. Although intention is a strong predictor of behavior according to the theory of planned behavior (Wang et al., 2024), there are well-known intention–behavior gaps in mental health contexts. Subsequent research should incorporate behavioral indicators—such as actual counseling attendance or digital platform usage—to more comprehensively evaluate the practical impact of malleability beliefs.

Finally, the study suggests promising intervention opportunities for enhancing malleability beliefs. Future research could draw upon digital intervention protocols from incremental theory studies (Crum et al., 2023; Yeager et al., 2022) to design scalable, technology-assisted programs tailored for college populations. Further work may also examine the long-term effects of such interventions and explore whether integrating GITT or Mindsponge-based elements (e.g., information-filtering exercises) can strengthen or accelerate belief change, stigma reduction, and help-seeking engagement.

## 7 Conclusion

This study demonstrated that beliefs about the malleability of mental health significantly predict Chinese college students’ intentions to seek on-campus psychological services, both directly and indirectly through reductions in help-seeking self-stigma. Depression severity further moderated the first stage of this pathway, weakening the stigma-reducing effect of malleability beliefs among students with higher depressive symptoms. These findings highlight the importance of fostering adaptive beliefs about mental health, reducing self-stigma, and providing differentiated support for students with varying levels of depression. Together, these results contribute to a deeper understanding of cognitive and emotional mechanisms underlying help-seeking and offer evidence-based directions for campus mental health interventions.

## Data Availability

The raw data supporting the conclusions of this article will be made available by the authors, without undue reservation.
